# Acknowledgment to Reviewers of *Biosensors* in 2021

**DOI:** 10.3390/bios12020080

**Published:** 2022-01-29

**Authors:** Biosensors Editorial Office

**Affiliations:** MDPI AG, St. Alban-Anlage 66, 4052 Basel, Switzerland

Rigorous peer-reviews are the basis of high-quality academic publishing. Thanks to the great efforts of our reviewers, *Biosensors* was able to maintain its standards for the high quality of its published papers. Thanks to the contribution of our reviewers, in 2021, the median time to first decision was 15 days and the median time to publication was 37 days. The editors would like to extend their gratitude and recognition to the following reviewers for their precious time and dedication, regardless of whether the papers they reviewed were finally published:
Abate, GiuliaLiou, Gunn‐GuangAbbas, NaseemLiu, ChaoxingAbreu, CarlosLiu, Cheng-HsienAdeloju, SamuelLiu, Chung-ChiunAdhikari, Bal RamLiu, FangAdhikari, Kishor KumarLiu, JiaAhire, Jayesh J.Liu, LuoAich, SatyabrataLiu, YifanAkhtar, JawaidLiu, YuAleksandrova, MariyaLiu, ZhifuAlfinito, EleonoraLivaniou, EvangeliaAlpar, DonatLobato-Morales, HumbertoAl-Sayah, MohammadLobo-Castañón, María JesúsAltini, MarcoLohr, DieterÁlvarez, María S.Londergan, CaseyAlvarez-Malmagro, JuliaLoo, Jacky F. C.Amorim, Cleber A.Lopes, PaulaAmouroux, MarineLopez Martinez, Maria JoséAnahtar, Melis N.López, Miguel ÁngelAnastassiu, HristosLópez-Martínez, AlbertoAndrianova, MariiaLorenzo, EncarnaciónAnni, MarcoLorenzo, JuliaAntunes, RuiLoyez, MédéricAoki, KazuhiroLu, DingqiangApetrei, ConstantinLucío, María IsabelAraújo, Alberto N.Luna Moreno, DonatoArsenin, Aleksey V.Luo, HaimingAsif, MuhammadLupu, StelianAyankojo, Akinrinade GeorgeLutz, DavidAydemir, NihanLyu, JianBadea Doni, MihaelaMa, GuangzhongBadea, MihaelaMa, LanBae, Hyung D.Ma, LinaBai, ShiMa, XingBajic, DraganaMacagnano, AntonellaBalvedi, Renata AlvesMacchia, EleonoraBañuelos, JorgeMaesako, MasatoBarek, JiříMaglio, OrnellaBaron, Julianne L.Magna, GabrieleBarrio, Melisa DelMalecka, KamilaBastos-Arrieta, JulioManderville, Richard A.Batrinou, AnthimiaMarcin Szymon, FilipiakBaumann, BerndMarconcini, PaoloBayram, AbdullahMarietta, FodorBeach, ChristopherMarpu, Sreekar BabuBelBruno, JosephMarques, Carlos Alberto FerreiraBene, LászlóMarrazza, GiovannaBercu, MioaraMartella, DanieleBeretta-Piccoli, MatteoMartin, Maria AlbaBerlina, Anna N.Martínez López, José IsraelBernasconi, RobertoMartínez-Lillo, JoséBerto, MarcelloMartínez-Periñán, EmilianoBhalla, NikhilMaruccio, GiuseppeBiagi, LyviaMaruthamuthu, MuraliBibikova, OlgaMaruthamuthu, MuralikannanBilatto, StanleyMarzullo, Vincenzo ManuelBing, TaoMascini, MarcelloBizzarri, Anna RitaMaskeliūnas, RytisBonyár, AttilaMastrangeli, MassimoBorges, Keyller BastosMathew, BobbyBorowik, PiotrMatos, Luís CarlosBortolotti, Carlo A.Matzeu, GiusyBossi, Alessandra MariaMauriz, ElbaBourguignon, NataliaMeder, RogerBourillot, EricMejía-Salazar, Jorge RicardoBratashov, Daniil N.Melnikov, PavelBrazaca, Laís CanniattiMendes, Renata KellyBrodnick, SarahMeng, LingjuBroecker, FelixMercado, Jesús M.Brouzgou, ΑngelikiMerrin, JackBrunetti, GiuseppeMi, XianqiangBuhot, ArnaudMicalizio, SalvatoreBukatin, Anton S.Michalski, JacekBunge, Christian-AlexanderMicheli, LauraBuoro, Rafael MartosMichelini, ElisaBurkin, MaximMiller, ElżbietaBusek, MathiasMiller, StephanieButt, Muhammad AliMin, JouhaBuzalewicz, IgorMin, MartCabaj, JoannaMin, RuiCai, JunMinerick, AdrienneCalin, BogdanMirasoli, MaraCalvo, BelenMishra, Geetesh K.Camerlingo, CarloMishra, RohitCanetta, ElisabettaMisiakos, KonstantinosCannistraro, SalvatoreMolin, DanielCanovas Martinez, RocioMondal, SudipCapelli, DavideMoon, Byeong-UiCardeñosa-Rubio, Maria CruzMoore, John H.Cardoso, FernandoMorales, AlejandroCarmo, João PauloMori, TetsushiCasals Terre, JasminaMostafa, Sheikh ShanawazCasquel Del Campo, RafaelMottet, DenisCastro, FláviaMrozek, PiotrCatarino, SusanaMu, XuanCaucheteur, ChristopheMuller, GerhardCavalera, SimoneMunteanu, Florentina-DanielaCésar Pereira, ArnaldoMurillo-Cabezas, FranciscoChai, ChanghoonMuroya, SusumuChakraborty, GoutamMurtada, KhaledChan, KannieNabok, AlexeiChan, Kiat HwaNagamine, KuniakiChancia, RobertNallal, MuthuchamyChang, Chia-ChenNam, Ki HyunChang, DingranNaqvi, RizwanAliChang, Seung-CheolNaroska, EdwinCharlton, Peter H.Nascimento, MicaelChavali, MurthyNasiri, RohollahChen, JinNasreddine, RoubaChen, JinghuaNath, Narayan Chandra DebChen, JuhongNegrea, PetruChen, LingxinNiemeyer, Christof M.Chen, PengyuNikitin, PetrChen, YanpingNikoleli, Dimitrios P.Cheng, JieNikolic, Maria VesnaCheng, Ji-YenNilghaz, AzadehChhetry, AshokNirala, Narsingh R.Chianella, IvaNishizawa, NozomiChiavaioli, FrancescoNoor, RadwanChiu, Nan-FuNovikov, Sergey M.Choi, Han-KyuNovikov, ValentinChoi, HeungjaeNovotný, FilipChoi, Jane RuNowak, RobertChoi, Jeong-WooNuster, RobertChoi, Jin-HaOcchicone, AgostinoChoi, JongchanOh, JonghyunChoi, JungilOlanrewaju, AyokunleChoi, Kyung HyunOlesberg, JonathonChoi, NamhyunOliveira, HelderChoi, SungyoungOliveira, Hugo M.Chon, KiOlyaee, SaeedCiampi, SimoneOmelyanchik, AlexanderCilli, EduardoÖpik, AndresCisterna, Daniel MarceloOrtega, Sara FrancoClark, Amanda M.Ortgies, Dirk H.Clark, Iain C.Osma, JohannClement, YuenOsminkina, LiubovCollings, NeilOsório, Jonas H.Concellón, AlbertoOsterman, NatanConniot, JoãoOstrovidov, SergeCordeiro, CristianoOza, GoldieCornelis, JanPaczkowski, JonCoros, MariaPalestri, PierpaoloCortés, Araceli GonzálezPalma, AlbertoCovington, JamesPaluch, EmilCrnkovic, AnaPan, HouwenCruz, AndreaPan, MatthewCubillana-Aguilera, LauraPankratov, DmitriiCui, FeiyunPannico, MariannaCui, KangPaolo, BollellaCumbo, CosimoPapazova, NinaCusso, FernandoParedes-Madrid, LeonelD’Acunto, MarioParia, DebadritaD’Andrea, CristianoPark, Chan HoD’Angelo, PasqualePark, ChulhwanD’Aurelio, RobertaPark, Hae-MinDa Silva Barros Allil, Regina CéliaPark, JaehoDabaghi, MohammadhosseinPark, Je-KyunDalamitros, AthanasiosPark, JongminDas, AnupamPark, Ki SooDas, PradipPark, MinDavid, Gabriela IuliaPatel, BrijeshkumarDe Geest, BartPatiño, YolandaDe Oliveira Junior, MarcosPavel, Ileana AlexandraDe Souza Gil, EricPedone, DeborahDe Stefano, LucaPeixoto De Almeida, MiguelDebela, Ahmed M.Pellitero, Miguel A.Deigner, Hans-PeterPeltomaa, RiikkaDel Pilar Taboada Sotomayor, MariaPeng, BoDelbarre Ladrat, ChristinePeng, ChunrongDelfino, InesPeng, HanyongDeLong, Robert K.Pereira, SoniaDe-los-Santos-Álvarez, NoemíPérez-Ràfols, ClaraDenisov, Ivan A.Perreault, JonathanDepciuch, JoannaPetelin, AndrejDhanjai, DhanjaiPéter, BeatrixDi Bartolomeo, AntonioPetrović, BojanDi Nardo, FabioPezeshki, AtiyeDias, DaianePezzolato, MarziaDíaz, Camilo A. R.Pfattner, RaphaelDiaz, PatriciaPiano, MartinaDiliën, HannePico, JesusDimaki, MariaPierella, CamillaDimitrova, LyudmilaPilan, LuisaDing, ShichaoPilát, ZdeněkDinu Gugoasa, Livia AlexandraPingarrón, JoséDominguez, BerenicePiro, BenoîtDominguez, Rocio B.Piskunov, SergeiDondajewska, EwelinaPitruzzello, GiampaoloDong, QiuchenPlatella, ChiaraDragonieri, SilvanoPodlipec, RokDubowski, Jan J.Podpora, MichalDuma, Virgil-FlorinPodwin, AgnieszkaDutra, Andeise CerqueiraPoenar, DanielDutta, AnushreePohanka, MiroslavDutta, GaurabPöhlmann, ChristopherDzantiev, Boris B.Polley, NabarunEbralidze, Iraklii I.Polo-Parada, LuisEersels, KasperPompa, Pier PaoloEl Nashar, Rasha MohamedPopescu, Ionel CatalinEleni, MakaronaPopov, AntonElsie, Parés-MatosPosada-Quintero, Hugo FernandoEnache, Teodor AdrianPospíšilová, MarieEscobedo, CarlosPrabowo, Briliant AdhiEspíndola, OtávioPrasad, AlishaEsposito, FlavioPrelipceanu, MariusEvtugyn, GennadyPriye, AashishFagadar-Cosma, EugeniaProchazka, MarekFan, YichongProctor, ChristopherFarka, ZdeněkPurohit, BuddhadevFateixa, SaraPurwidyantri, AgnesFebbraio, FerdinandoPushpavanam, KarthikFei, YiyanPustan, MariusFeng, ShilunQian, ChunqiFeng, ShuanglongQin, PeiwuFerens-Sieczkowska, MirosławaQing, Lin-SenFerhan, Abdul RahimQiu, GuangyuFerrández Vicente, José ManuelRabbani, GulamFerrari, Alejandro Garcia-MirandaRabiee, NavidFerraro, DavideRahimi, ParvanehFerreira, MarystelaRahman, Md JuberFigueira, FlavioRaimundo, Armando Manuel De MendonçaFilippeschi, AlessandroRamos, DanielFiorillo, AntoninoRamos, Marco A.Firtat, BogdanRay, SujayFisher, Aron B.Raymundo-Pereira, Paulo A.Florescu, MonicaRazumiene, JulijaFoguel, Marcos ViniciusReale, RiccardoFologea, DanielReddy, Mogalahalli V.Fortunati, IlariaRengarajan, VenkatakrishnanFraleoni-Morgera, AlessandroRichtera, LukášFrancés-Soriano, LauraRighetti, LauraFranco, Diego LeoniRizal, ConradFranco, DomenicoRizzo, CarmenFrank, Ludmila A.Robinson, MathewFrense, DieterRocchitta, GaiaFu, GengtaoRodrigues-de-Souza, Daiana PriscilaFurlan De Oliveira, RafaelRodriguez-Lorenzo, LauraGaft, MichaelRoka-Moiia, YanaGalan-Vidal, Carlos AndresRomero-Sánchez, FranciscoGalian, Raquel E.Rothkrantz, LeonGallelli, LucaRoy, DhruvajyotiGamarra, Lionel FernelRubin, Robert L.Gao, ShiqiangRutyna, IwonaGarcía-Meza, Jessica ViridianaRyazantsev, MikhailGaroli, DenisSadabadi, HamidGartia, Manas RanjanSadok, IlonaGąsior-Głogowska, MarlenaSaftics, AndrasGasiūnas, GiedriusSaharan, LalitaGauden, PiotrSahu, RakeshGe, GuangboSahu, Rama ShankerGerislioglu, BurakSaito, NorikoGheorghiu, EugenSakthivel, KogularasuGhiami, AmirSallabi, Farag M.Ghibaudo, GérardSamiei, EhsanGhita, AdrianSang, MeiGillanders, Ross N.Santana, TedGiménez, DavidSantilli, ManlioGioulekas, FotiosSantos, AdrianoGnyba, MarcinSantos, Jaime Batista DosGodinho, Maria HelenaSantos, João H. P. M.Gómez-Graña, SergioSaratale, GaneshGong, ShuSarolic, AntonioGong, ZhenfengSasaki, AkiraGonzález-Cortés, AraceliSawan, MohamadGonzález-González, MirnaSbrollini, AgneseGöpfert, BeatScaramuzza, MatteoGopu, Sriram GopuSchulze-Tanzil, Gundula GesineGorodkiewicz, EwaSchwenker, FriedhelmGrabowska, IwonaSedgwick, Adam C.Grossi, MarcoSedlackova, EliskaGruić, IgorSeibold, Gerd MichaelGrzegorz, Nałȩcz-JaweckiSempionatto, Juliane R.Gualandi, IsaccoSenel, MehmetGüell, MarcSensi, MatteoGui, LinSergeyev, VladimirGuieu, SamuelSerrano-Pertierra, EstherGul, IjazSerry, MohamedGumuscu, BurcuServi, MichaelaGuo, QingleiSessa, FrancescoGupta, SanjuShah, PratikGurgu, Leontina C.Sharafeldin, MohamedGut, KazimierzSher, MazharGutiérrez, Juan ManuelSherratt, R. SimonGuzmán, EduardoShevtsov, MaximHacker, Mallory L.Shin, Dong-SikHaddad, RafiShokouhimehr, MohammadrezaHaghi, MostafaShrirao, AnilHansen, ClintShrivastav, Anand MohanHara, MasanoriShtepliuk, IvanHarpaz, DorinShumilov, EvgeniiHatamie, AmirSiavash, RoozbehHerkendell, KatharinaSierra Rosales, PaulinaHessinger, CarolinSignore, GiovanniHianik, TiborSilva, Everson Thiago Santos GerôcioHida, Richard YudiSilveira, CéliaHirtz, MichaelSingh, Narendra K.Hong, Ka L.Singha, SubhankarHong, Kyeong-ManSinha-Ray, SumanHong, Sang GiSinibaldi, AlbertoHonzíček, JanSistla, SrinivasHorn, IvoSkládal, PetrHorvath, RobertSkliarova, IouliiaHossain, Md. FarukSkorucak, JelenaHosu, OanaSkvortsov, Alexey N.Hsieh, KuangwenSlodek, AnetaHsu, Ching-ChiSmarandache, AdrianaHsu, Huan-HsuanSobiech, MonikaHuang, CanSojic, NesoHuang, ChenyuSokolikova, MariaHuang, Chia-ChiSokullu, EsenHuang, ChiayiSoldado-Cabezuelo, Ana BelénHuang, Chih-ShengSolovieva, AnastasiyaHudry, DamienSong, ShipingHumbert, GeorgesSonia, GeraHuptych, MichalSoto Rodriguez, Paul Eduardo DavidIáñez, EduardoSpagnuolo, GianricoImtiaz, MasudulSpanedda, MariaIndic, PremanandaSpolaor, FabiolaIoan, TudosaSqueo, GiacomoIonete, Eusebiu IlarianStaiano, MariaIrvine, Gordon W.Stephens, Andrew F.Isokawa, TeijiroStokke, Bjørn TorgerIvanišević, IrenaStortini, AngelaJacob, Maik H.Strianese, MariaJaqueline Kaschuk, JoiceStrobbia, PietroJasielec, Jerzy J.Su, FengxiaJemere, Abebaw BelaySu, JudithJenkins, DavidSui, ShuoJi, WeiSukeri, AnandhakumarJia, KunSukhavasi, Susrutha BabuJiang, ChaozheSulej, JustynaJiang, HongjieSun, DuanpingJiang, ShanSzlósarczyk, MarekJiang, YongSzot-Karpińska, KatarzynaJilani, AsimTa, Hang ThuJiménez-Espinoza, CarmenTalaikis, MartynasJiménez-Pérez, RebecaTanasova, MarinaJohari, MohammadTangirala, Venkata Krishna KarthikJozanović, MarijaTasca, FedericoJuan, Carlos G.Tay, Jian WeiJurinjak Tusek, AnaTaylor, Thomas MatthewKakoti, AnkanaTeodorescu, FlorinaKalkan, A. KaanThakur, RavirajKamel, Ayman H.Thiruppathi, Antony R.Kämpfer-Homsy, AlexandraTian, KeKana, AntoninTibbetts, Katharine MooreKang, ChanKyuTjioe, MarcoKant, KrishnaTkac, JanKarkowska-Kuleta, JustynaToldrà, AnnaKashima, ShinTombelli, SaraKatz, EvgenyTonello, SarahKawakami, JunTorcates, Lourdes RivasKeller, AdrianTorres-Torres, CarlosKerman, KaganTrontelj, JanezKeshavarz, MeysamTsagkaris, AristeidisKhalid, AsmaTsigaridas, Georgios N.Khan, RayyanTsunoda, MakotoKim, Chi-JuTsutsui, MakusuKim, DaeEunTyagi, MuditKim, JeesuUrbančič, IztokKim, Jin YoungUrbanek-Trzeciak, Martyna O.Kim, Nam-YoungUykur, EceKim, SamVacacela Gomez, CristianKim, SeunghyunValsecchi, ChiaraKinet, DamienVan De Walle, AuroreKintzios, SpyridonVan Leeuwen, HansKiourtis, AthanasiosVarodi, CodrutaKitaguchi, TetsuyaVedrine, ChristopheKitahama, YasutakaVelázquez, RamiroKitamura, AkiraVenkatasubramanian, AnandramKitazumi, YukiVernick, SefiKlueh, UlrikeVesin, Jean-MarcKnapkiewicz, PawelVestergaard, Mun’delanji C.Kokkoris, GeorgeVidic, JasminaKong, Kien VoonVillarreal-Gómez, Luis JesúsKoo, Kevin M.Viveros‐Ceballos, José LuisKorri-Youssoufi, HafsaVon Neuhoff, NilsKost, BartłomiejVrba, JanKowalczyk, AgataWales, DominicKozitsina, AlisaWang, BoKrawczynski, KamilWang, DaliKrebsz, MelindaWang, Hsiang-ChenKubinyi, MiklósWang, LeiKukushkin, VladimirWang, LihuaKulbacka, JulitaWegener, JoachimKulkarni, TanmayWei, JingKumar, AbhishekWeinzierl, RobertKumar, ShailabhWieclaw, LukaszKuncová, GabrielaWischhusen, JenniferKunrath, Marcel F.Witkowska Nery, EmiliaKunsági-Máté, SándorWójcik, DariuszKurita, KazunariWon, Sang M.Kurlyandskaya, Galina V.Wong, Man-kinKusior, AnnaWorwag, MalgorzataKuttner, ChristianWrobel, PiotrLaabei, MaisemWu, DiLafrenie, Robert M.Wu, GuangfuLaguarda-Miró, NicolásWu, JayneLai, StefanoWu, LongLaiwalla, FarahWu, Shu-PaoLáng, Győző G.Xiong, ZeLang, Hans PeterXu, FengLaranjo, MafaldaXu, JingjingLaskowska, MagdalenaXu, KaikaiLaurenti, MarcoYagati, Ajay KumarLe Faucheur, AlexisYang, DemingLe Thai, DuyYang, LetaoLe, Nguyen Quoc KhanhYang, QuansanLee, Chang-SeukYang, ShujieLee, Hyuck JinYarman, AysuLee, Hyun HoYen, Feng-LinLee, HyungseokYilmaz, MehmetLee, JaewookYim, ChangyongLee, Jeong SuYokota, KazumichiLee, Kyoung G.Yoneyama, HiroshiLee, Min-HoYoon, JinhoLee, Seok JaeYoshihara, EijiLee, Seung HwanYu, ChenxuLee, TaekYu, ShudongLentz, ChristianYusof, Nor AzahLeon-Juarez, MoisesZaidi, Shabi AbbasLesiak, PiotrZamfir, Lucian-GabrielLevine, MindyZanchetta, GiulianoLeyva-Gómez, GerardoZarrin, HadisLi, GuangliZavala-Rivera, PaulLi, JieZdarta, AgataLi, JinZendrini, AndreaLi, PengZeng, ShuwenLi, QinceZhang, JinyangLi, YangZhang, JuanLi, YingchunZhang, LiweiLi, ZhengxinZhao, JingyiLiang, GangZhou, HaiboLiao, Jiunn-DerZhou, JianLima, IvanZhou, Xiao DongLin, Cheng-TeZimmer, MarcLin, Shin-PingZiółkowski, RobertLindemann, StephanZolotykh, Nikolai YuLindquist, NathanZucca, ClaudioLinek, PawełZuffo, MichelaLingwood, DanielŻyczkowski, Marek


